# Characterization of the rock pigeon type IV interferon system reveals conserved tyrosine-based signaling via the IFN-υR1/IL10RB complex

**DOI:** 10.1186/s13567-026-01823-w

**Published:** 2026-08-12

**Authors:** An Ning Pang, Jia Yi Li, Shan Nan Chen, Xiang Yang Wu, Nan Li, Lan Hao Liu, P. Nie

**Affiliations:** 1 School of Marine Science and Engineering, Qingdao Agricultural University, Qingdao, 266109 Shandong Province China; 2 State Key Laboratory of Breeding Biotechnology and Sustainable Aquaculture and Key Laboratory of Aquaculture Disease Control, Institute of Hydrobiology, Chinese Academy of Sciences, Wuhan, 430072 Hubei Province China

**Keywords:** Type IV IFN, IFN-υ, Rock pigeon, IFN-υR1, IL10RB

## Abstract

Type IV interferon (IFN), a recently identified class of IFN, exhibits broad-spectrum antiviral and antibacterial functions across species. In this study, the type IV IFN gene, *IFN*-*υ*, and its cognate receptor subunits (IFN-υR1 and IL10RB) were functionally characterized in an avian species, the rock pigeon (*Columba livia*). Phylogenetic analysis showed that rock pigeon IFN-υ, IFN-υR1, and IL10RB were clustered closely with their respective orthologs in other vertebrates. Expression profiling revealed the constitutive transcription of these genes across multiple tissues. Furthermore, the expression of these genes was significantly upregulated upon stimulation with the viral double-stranded RNA (dsRNA) mimic, poly(I:C). The recombinant rock pigeon IFN-υ activated the canonical Janus kinase–signal transducer and activator of transcription (JAK-STAT) signaling pathway, inducing STAT1 phosphorylation and the consequent expression of key interferon-stimulated genes (ISGs) such as *OAS1*, *PKR,* and *RSAD2*. Using overexpression and knockdown assays, the biological activity of IFN-υ was found to signal through a heterodimeric receptor complex comprising IFN-υR1 and IL10RB. Importantly, two conserved tyrosine residues (Tyr_334_ and Tyr_525_) within the intracellular domain of IFN-υR1 were found essential for IFN-υ-mediated JAK-STAT pathway activation, ISG induction, and antiviral activity. These findings elucidate the conserved functional architecture and signaling mechanism of type IV IFN in vertebrates.

**Supplementary Information:**

The online version contains supplementary material available at 10.1186/s13567-026-01823-w.

## Introduction

Interferons (IFNs) constitute a pivotal family of cytokines central to vertebrate defense against pathogens [1, 2]. They are currently classified into four types—I, II, III, and IV—which collectively form a multilayered and cooperative defense network through specific receptor systems and shared or divergent signaling pathways [3–6]. Type I IFNs (e.g., IFN-α/β), the most diverse subgroup, signal through the IFNAR1/IFNAR2 receptor complex to rapidly induce the expression of hundreds of interferon-stimulated genes (ISGs), establishing a broad and systemic antiviral state [7, 8]. Type II IFN (IFN-γ) acts primarily via the IFNGR1/IFNGR2 receptor to activate immune cells, thereby bridging innate and adaptive immunity [6, 9]. Type III IFNs (IFN-λ) utilize the IL10RB/IFNLR1 receptor complex, and their response is largely restricted to epithelial and mucosal barriers, providing localized and sustained defense [10, 11]. Type IV IFN (IFN-υ), an evolutionarily ancient group present in many mammals, birds, reptiles, amphibians, and fish, despite its absence in human genome, signals through a specific receptor complex primarily composed of IFN-υR1 and IL10RB via the Janus kinase–signal transducer and activator of transcription (JAK-STAT) pathway [3, 5, 12].

Based on its unique evolutionary status and activity, research on the function of IFN-υ has primarily been conducted in non-mammalian vertebrates where it remains functional—especially in fish and amphibians [5]. Recent studies have confirmed its potent antiviral and antibacterial functions and, in parallel, gradually revealed its specific mechanisms of action. Evidence from teleost fish demonstrates its broad antiviral activity: in zebrafish (*Danio rerio*), IFN-υ can significantly inhibit the infection by grass carp reovirus 2 (GCRV-II) [5]; in black carp (*Mylopharyngodon piceus*), it can upregulate the expression of ISGs such as *Mx1*, *PKR*, *ISG15*, and *RSAD2*, thereby enhancing the host cell resistance to spring viremia of carp virus (SVCV) replication [13]. Similarly, in blunt snout bream (*Megalobrama amblycephala*), IFN-υ responds to poly(I:C) induction and promotes the expression of multiple antiviral-related ISGs [14]. Even in more evolutionarily ancient cartilaginous fish such as the white spotted bamboo shark (*Chiloscyllium plagiosum*), IFN-υ remains inducible by poly(I:C), suggesting evolutionary conservation of its immune induction mechanism [4]. At the molecular level, studies in crucian carp (*Carassius gibelio*) reveal that IFN-υ binds to the CRFB4/CRFB12 receptor complex, activating not only the classical JAK-STAT pathway but also mitogen-activated protein kinase (MAPK) and phosphatidylinositol 3-kinase (PI3K) signaling cascades, which collectively induce ISG expression and effectively inhibit SVCV infection [3]. In addition to the knowledge obtained in fish, IFN-υ also plays a protective role in amphibians, as reported in the clawed frog (*Xenopus laevis*), where it significantly inhibits frog virus 3 (FV3) infection [5, 12]. On the other hand, type IV IFN can contribute substantially to antibacterial immunity; for example, in grass carp (*Ctenopharyngodon idella*), it provides defense against both Gram-negative and Gram-positive bacterial infections [15].

The canonical signaling cascade across all IFN types converges on the JAK-STAT pathway, which orchestrates ISG transcription through a conserved architectural blueprint: the recruitment of specific STAT proteins to phosphorylated tyrosine motifs within receptor cytoplasmic domains [16–19]. This mechanism is definitively established for type I, II, and III IFN signaling. It is illustrated by STAT2 docking at Tyr_466_ of IFNAR1 and, in type I responses [20–22], by STAT2 docking at Tyr_337_ and Tyr_512_ of IFNAR2c as well as by STAT1 docking at Tyr_337_ and Tyr_512_ of IFNAR2c [23]. Parallel paradigms include the indispensable role of IFNGR1 Tyr_440_ for STAT1 activation in type II IFN signaling [18, 24], and in type III signaling, by STAT2 recruitment to both Tyr_343_ and Tyr_517_ on IFN-λR1, and by STAT1 recruitment to Tyr_517_ on IFN-λR1 [25]. Similarly, type IV IFN is believed to signal through a heterodimeric receptor, where ligand-induced JAK-mediated phosphorylation is expected to create docking sites preferentially for STAT1 and STAT2, leading to their phosphorylation, dimerization, and nuclear translocation, and ultimately the formation of transcriptional complexes such as ISGF3 with interferon regulatory factor 9 (IRF9) [3, 12]. However, in contrast to the well-defined pathways of other IFNs, key aspects of type IV IFN signaling, particularly its precise receptor composition, the identity of critical phosphotyrosine docking sites, and the detailed organization of its JAK-STAT axis, remain unresolved in many species, representing a crucial area to be elucidated for this immune pathway.

In this study, the IFN-υ gene and its possible receptor subunits, IFN-υR1 and IL10RB, were identified in rock pigeon *Columba livia*, and the antiviral activity of rock pigeon IFN-υ was further investigated in suppressing Tembusu virus replication in host cells. To further dissect the molecular basis of this functional initiation, sequence alignment analysis was performed to identify the possible tyrosine residues within the intracellular domain of IFN-υR1, and through site-directed mutagenesis and co-immunoprecipitation (Co-IP) experiments, the role of two tyrosine residues in the formation of STAT docking sites was examined, and the subsequent activation of downstream JAK-STAT pathway and induction of ISG expression by rock pigeon type IV IFN were further revealed in this study. These findings provide a critical molecular basis for understanding type IV IFN signal initiation in birds, while also providing clues to the evolutionary conservation of this signaling module in vertebrates.

## Materials and methods

### Animals and cell lines

The rock pigeons used in this study were obtained from a breeding farm in Hubei Province, China, and housed in isolators with commercial feed. Human embryonic kidney 293 T (HEK293T) cells were obtained from the American Type Culture Collection (ATCC, CRL-3216) and were routinely cultured in Dulbecco’s modified Eagle’s medium (DMEM) (Gibco, C11995500BT) supplemented with 10% fetal bovine serum (FBS) (Vazyme, F102-01) at 37 °C in a humidified atmosphere incubator containing 5% CO_2_.

Pigeon and duck embryonic fibroblasts (PEFs and DEFs, respectively) were isolated from 9- to 11-day-old embryos. The PEFs and DEFs were maintained in DMEM supplemented with 10% FBS and 1% penicillin–streptomycin (PS) (HyClone, SV30010), and also incubated at 37 °C in a humidified atmosphere containing 5% CO_2_.

### Antibodies and siRNA primers

Commercial primary and secondary antibodies include Flag-Tag (Sigma-Aldrich, F1804, 1:6000 dilution), HA-Tag (Cell Signaling Technology, 3724S, 1:5000 dilution), STAT1 (ABclonal Technology, A19563, 1:5500 dilution), β-actin (ABclonal Technology, AC026, 1:20000 dilution), phospho-STAT1 (Sangon Biotechnology, D155017, 1:4500 dilution), anti-GFP (green fluorescent protein) (ABclonal Technology, AE078, 1:6000 dilution), horseradish peroxidase (HRP)-conjugated anti-Flag (AlpVHHS, 016–303-005, 1:5000 dilution), goat anti-rabbit (Invitrogen, 31460, 1:6000 dilution), and goat anti-mouse (Abcam, ab6789, 1:8000 dilution).

The small interfering RNA (siRNA) primers targeting rock pigeon IFN-υR1 (sense: GGACAGAACCUCAGUGUAA) and IL10RB (sense: GGCUCAUGGAAUUACAGAA), synthesized by Tsingke Biotechnology (Beijing, China), were transfected into PEFs using the TransIT-X2^®^ delivery system (Mirus Bio, MIR6000), with transfection efficiency determined by quantitative real-time fluorescence polymerase chain reaction (qRT-PCR).

### Quantitative real-time fluorescence PCR (qRT-PCR)

Total mRNA was extracted from organs/tissues, DEFs, and PEFs using the VAMNE Magnetic Cell/Tissue Total RNA Kit (Vazyme, RMA101-C2-P3). The RNA was then reverse-transcribed into cDNA using the HiScript III All-in-one RT SuperMix (Vazyme, R333-01) according to the manufacturer’s instruction. Finally, qRT-PCR was performed on a QuantStudio 3 Real-Time PCR System with the SupRealQ Purple Universal SYBR qPCR Master Mix (Vazyme, Q412-03). The relative expression level of ISGs was calculated via the 2^−ΔΔCt^ method [26], using *GAPDH* as the endogenous control for normalization [27]. The primer sequences used for this part are provided in Table 1.

**Table 1:** **PCR primers used in this study**

Primer	Sequence 5’–3’	Usage
IFN-υ-Flag-F	TAAGCTTGCGGCCGCGAATTCATGTATTACCCTGGGAGCTTC	Eukaryotic expression
IFN-υ-Flag-R	TCTAGAGTCGACTGGTACCGAATGTCTTGCCCTTCTGAAAG	
IFN-υR1-Flag-F	TAAGCTTGCGGCCGCGAATTCATGGGGTGCAGAGGGATGA	
IFN-υR1-Flag-R	CTCTAGAGTCGACTGGTACCGACAGCTCACTGGTAACCGGG	
IL10RB-Flag-F	TAAGCTTGCGGCCGCGAATTCATGGCCGCCGCCCTCCGC	
IL10RB-Flag-R	CTCTAGAGTCGACTGGTACCGAGGCTTTTGATGAAGCTGGTA	
IFN-υR1-mut1-inF	CAGGACACTAGTGGC**TTC**TGTGCCAACGGGTTTGGG	
IFN-υR1-mut1-inR	CCCAAACCCGTTGGCACAGAAGCCACTAGTGTCCTG	
IFN-υR1-mut23-inF	AGAGACAGC**TTC**ACAGGTGACAGGGAC**TTC**CGGACATCC	
IFN-υR1-mut23-inR	GGATGTCCGGAAGTCCCTGTCACCTGTGAAGCTGTCTCT	
IFN-υR1-mut4-inF	CTGCACCTCCAGCTT**TTT**TCTAAATGCCAGTGCCCG	
IFN-υR1-mut4-inR	CGGGCACTGGCATTTAGAAAAAAGCTGGAGGTGCAG	
IFN-υR1-mut5-inF	TCCGGC**TTC**GAGCTGCGGCCCCCGGTTACCAGTGAGCTG	
IFN-υR1-mut5-inR	CAGCTCACTGGTAACCGGGGGCCGCAGCTCGAAGCCGGA	
YFFFF-inF	CAGGACACTAGTGGC**TAC**TGTGCCAACGGGTTTGGG	
YFFFF-inR	CCCAAACCCGTTGGCACAGTAGCCACTAGTGTCCTG	
FYFFF-inF	AGAGACAGCTACACAGGTGACAGGGACTTCCGGACATCC	
FYFFF-inR	GGATGTCCGGAAGTCCCTGTCACCTGTGTAGCTGTCTCT	
FFYFF-inF	AGAGACAGCTTCACAGGTGACAGGGAC**TAC**CGGACATCC	
FFYFF-inR	GGATGTCCGGTAGTCCCTGTCACCTGTGAAGCTGTCTCT	
FFFYF-inF	CTGCACCTCCAGCTT**TAT**TCTAAATGCCAGTGCCCG	
FFFYF-inR	CGGGCACTGGCATTTAGAATAAAGCTGGAGGTGCAG	
FFFFY-inF	TCCGGC**TAC**GAGCTGCGGCCCCCGGTTACCAGTGAGCTG	
FFFFY-inR	CAGCTCACTGGTAACCGGGGGCCGCAGCTCGTAGCCGGA	
ClHA-STAT1-F	TTCCTGCAGGATATCAAGCTTATGGCTCAGTGGTATCAGCTC	
ClHA-STAT1-R	GAGGTCGACGGATCCGGTACCTTACGTTGTATATGCTGAACA	
ClRSAD2-pro-F	CAGTACCGGAATGCCAAGCTTAGATGCACAATTTGAAGTGTT	
ClRSAD2-pro-R	TCTTACGCGTGCTAGCCCGGGGGCTCGGCTCGATCTGTTT	
ApHA-STAT1-F	TTCCTGCAGGATATCAAGCTTATGGCCGCGGCCCGGCGG	
ApHA-STAT1-R	GAGGTCGACGGATCCGGTACCTTAAGTTGAATATGCTGAA	
Ap-IL10RB-GFP-F	CTACCGGACTCAGATCTCGAGATGGCCGCCGCCCTGCGG	
Ap-IL10RB-GFP-R	CCGTCGACTGCAGAATTCGGTCCCTTTCTGAAGCTGGTAATA	
qApβ-actin-F	CCGGGCATCGCTGACA	Real-time PCR
qApβ-actin-R	GGATTCATCATACTCCTGCTTTGCT	
qApMx-F	AAAGACGGTGGGAAGGAGAT	
qApMx-R	TGAAGGACAGCAGATAGGATGA	
qApOASL-F	TCTTCCTCAGCTGCTTCTCC	
qApOASL-R	ACTTCGATGGACTCGCTGTT	
qApRSAD2-F	GCCCTGAGAGAAGCAGAGAA	
qApRSAD2-R	AAGGCTCTTTCCGTCCATTT	
qClGAPDH-F	ATGACCACTGTCCATGCTATC	
qClGAPDH-R	GCTCCAGTAGATGCTGGAATAA	
qClIFN-υ-F	ACCCTGGGAGCTTCTTCTTC	
qClIFN-υ-R	GCTCACTTCCTCACACTCCA	
qClIFN-υR1-F	TACATCTTCCCCAGCCCATC	
qClIFN-υR1-R	GTAGCCACTAGTGTCCTGGG	
qClIL10RB-F	CGAGCAGACAACCCACAATG	
qClIL10RB-R	GCTCTTCTTGTGGGAGTGGA	
qClOAS1-F	GCTGATACTGCTGCACACC	
qClOAS1-R	TTATTCCAGCTTTCCTTGGAGC	
qClPKR-F	GACTTCGGTCTTGTGACTTCTC	
qClPKR-R	GACTGTTCTGGTGCCATGTAT	
qClRSAD2-F	AGACTTCGAACGATTCCTGG	
qClRSAD2-R	ACTCCACACATACTTTCCTCC	
qClSTAT1-F	TCTCTGCTGTTACTTTCCCTG	
qClSTAT1-R	CGAGAGACCTCGTAGAATTC	
qDTMUV-E–F	AATGGCTGTGGCTTGTTTGG	
qDTMUV-E-R	GGGCGTTATCACGAATCTA	

*Cl* represents *Columba livia*, and Ap *Anas platyrhynchos*.

### Plasmid construction and transfection

The full-length open reading frames (ORF) of rock pigeon IFN-υ, IFN-υR1, IL10RB, and IFN-υR1 variants were cloned into the p3xFlag-cmv-14 expression vector to generate 3× Flag-tagged constructs. The ORF of duck IL10RB was subcloned into the pEGFP-N1 vector to generate a GFP-tagged construct. For duck and rock pigeon STAT1, the respective ORF was subcloned into the pcDNA3.1-HA vector to create HA-tagged expression plasmids. In addition, a 2.0-kilobase (kb) promoter region (positions −1822 to +178) of the rock pigeon *RSAD2* gene was PCR-amplified and subcloned into the firefly luciferase reporter vector pGL3-Basic. All expression plasmids were constructed in existing vectors via homologous recombination using the ClonExpress II One Step Cloning Kit (Vazyme, C112-02). The integrity of all constructs was verified by sequencing (Sangon Biotechnology). All primer sequences used for plasmid construction are provided in Table 1.

The constructed plasmids were then transfected into HEK293T cells or DEFs and PEFs using ExFect™ Transfection Reagent (Vazyme, T101-02), in accordance with the manufacturer's instructions. Briefly, cells at 70–80% confluency were transfected with plasmid DNA-reagent complexes prepared in opti-MEM. The DNA-to-reagent ratio was 1 µg/2 µL for each cell type based on preliminary titration experiments. Transfection efficiency was routinely monitored by parallel transfection with an EGFP-expressing control vector.

### Isolation of leukocytes from rock pigeon liver

A healthy adult rock pigeon was euthanized and surface-sterilized by immersion in 75% ethanol for 5 min. Under aseptic conditions in a laminar flow hood, the liver was excised and placed in a dish containing ice-cold phosphate-buffered saline (PBS). The tissue was minced into fine pieces and gently ground through a 100 μm cell strainer with an appropriate volume of PBS. The resulting filtrate was further passed through a 200 μm strainer to remove tissue debris. The cell suspension was centrifuged at 500 × *g* for 5 min at 4 °C, the supernatant was discarded, and the pellet was resuspended in cold PBS.

To further purify leukocytes, a discontinuous Percoll (Sigma-Aldrich, P1644-100 mL) density-gradient centrifugation was performed. Briefly, 3 mL 51% Percoll solution (prepared in 1× PBS, pH 7.2–7.4) was carefully underlaid with 3 mL 34% Percoll solution in a 15 mL centrifuge tube to form a clear two-layer gradient. The prepared cell suspension was then gently layered on top of the gradient. Centrifugation was carried out at 800 × *g* for 30 min at 4 °C with the lowest acceleration and without brake. After centrifugation, the leukocyte layer located at the 34%/51% Percoll interface was carefully aspirated and transferred to a new tube. Cells were washed twice with an excess of PBS (centrifugation at 500 × *g* for 10 min each) to remove residual Percoll. Finally, leukocytes were resuspended in complete DMEM supplemented with 10% FBS and 1% PS, counted, and adjusted to desired concentrations for subsequent experiments.

### Production of recombinant rock pigeon IFN-υ protein

To obtain rock pigeon IFN-υ recombinant protein, HEK293T cells were transfected with the IFN-υ-Flag expression construct. Forty-eight hours post-transfection, the culture supernatant was collected as the source of the recombinant protein, with expression confirmed by Western blotting. The harvested protein was then applied to isolated primary cells at a working concentration of 100 ng/mL. At indicated time points post-stimulation, cell samples were collected to assess the activation of the JAK-STAT signaling pathway and the subsequent induction of ISGs.

### Viral infection and measurement of viral titer

The duck Tembusu virus (DTMUV) CQW1 strain (GenBank accession number: KM233707.1) was maintained in a laboratory in Sichuan Agricultural University and propagated in DEFs [28].

DEFs were cultured in 24-well plates until reaching 70–80% confluency. The cells were transfected with different combinations of target genes (or corresponding vectors as controls) using ExFect^™^ Transfection Reagent. At 24 h post-transfection, DEFs were pretreated with recombinant IFN-υ for 24 h, followed by infection with DTMUV at a multiplicity of infection (MOI) of 0.2. Following 1 h viral adsorption, the inoculum was removed and replaced with fresh 2% FBS DMEM. Cells were further incubated for 40 h before analysis.

Viral titers were determined by a tissue culture infectious dose (TCID_50_) assay using serially diluted culture supernatants. In parallel, total mRNA was extracted from infected cells, and DTMUV copy numbers were quantified by qRT-PCR using primers specific for the DTMUV *E* gene (DTMUV-*E*) [29]. All experiments were performed with three independent biological replicates.

### Luciferase reporter assay

PEFs were first transfected with siRNA targeting IFN-υR1 and IL10RB, respectively, at a final concentration of 50 nM for 48 h. Afterwards, cells were transfected with the RSAD2-promoter reporter construct for another 24 h and then treated with recombinant IFN-υ overnight. Subsequently, cells were lysed in 100 µL of Passive Lysis Buffer (Promega, E1941) for 30 min at room temperature (RT). After centrifugation to remove debris, luciferase activity was measured in 10 µL supernatant using Luciferase Assay Reagent (Promega, E1960). Luminescence measurements were obtained with a GloMax^®^ Multi Jr system (Promega) and normalized to the internal Renilla luciferase signal.

### Western blotting and co-immunoprecipitation

Western blotting was performed as described in previous study [30]. In brief, fully lysed protein samples from cells were mixed with 5× loading buffer supplemented with 10% β-mercaptoethanol, heated at 100 °C for 10 min, and then separated by 8%, 10%, or 12% sodium dodecyl sulfate–polyacrylamide gel electrophoresis (SDS-PAGE) under reducing conditions. At the end of electrophoresis, the separated protein samples were electroblotted onto a 0.45 µm polyvinylidene difluoride membrane (Merck Millipore, IPVH00010) and blocked for 1.5 h at room temperature in tris-buffered saline (TBS) (10 mM Tris–HCl, pH 8.0, containing 150 mM NaCl) containing 5% skim milk and 0.1‰ Tween 20. Primary antibody was diluted with 1% skim milk for incubation overnight at 4 °C. In the following day, the blot was washed three times with TBS containing 0.1‰ Tween 20 and incubated with the appropriate horseradish peroxidase (HRP)-conjugated secondary antibody for 1.5 h at RT. Finally, HRP binding to secondary antibodies was detected in a ChemiDoc™ MP Imaging System (Bio-Rad) using an ultrasensitive chemiluminescence (ECL) kit (Merck Millipore, WBKLS0500).

Protein–protein interactions were analyzed by co-immunoprecipitation (Co-IP). DEFs or PEFs were transfected with the indicated plasmids and lysed in Pierce™ IP lysis buffer (1 mM EDTA, 150 mM NaCl, 25 mM Tris–HCl pH 7.4, 1% NP-40, 5% glycerol) (Thermo Fisher Scientific, 87788) containing Halt™ Protease and Phosphatase Inhibitor Cocktail (Thermo Fisher Scientific, 78442). After centrifugation at 2000 × *g* for 10 min to remove debris, 10% of the supernatant was reserved as input. For Flag-tagged protein immunoprecipitation, the remaining supernatant were incubated overnight with anti-Flag M2 affinity gel (Sigma-Aldrich, A2220). The immunoprecipitates were washed five times with IP lysis buffer, followed by elution in SDS-PAGE loading buffer at 100 °C for 10 min. Eluted proteins were then analyzed by Western blotting.

### Statistical analysis

Statistical analyses were conducted using SPSS 22.0 or GraphPad Prism 9 software. Data are presented as mean ± standard error (SE). Differences between groups were evaluated using unpaired Student's t-test or one-way analysis of variance (ANOVA), as appropriate. Statistical significance was defined as *P < 0.05, **P < 0.01, or ns for nonsignificant results. Details of specific statistical tests used are provided in the respective figure legends.

## Results

### Sequence characterization and phylogenetic analysis of rock pigeon IFN-υ, IFN-υR1, and IL10RB

**Figure 1:**
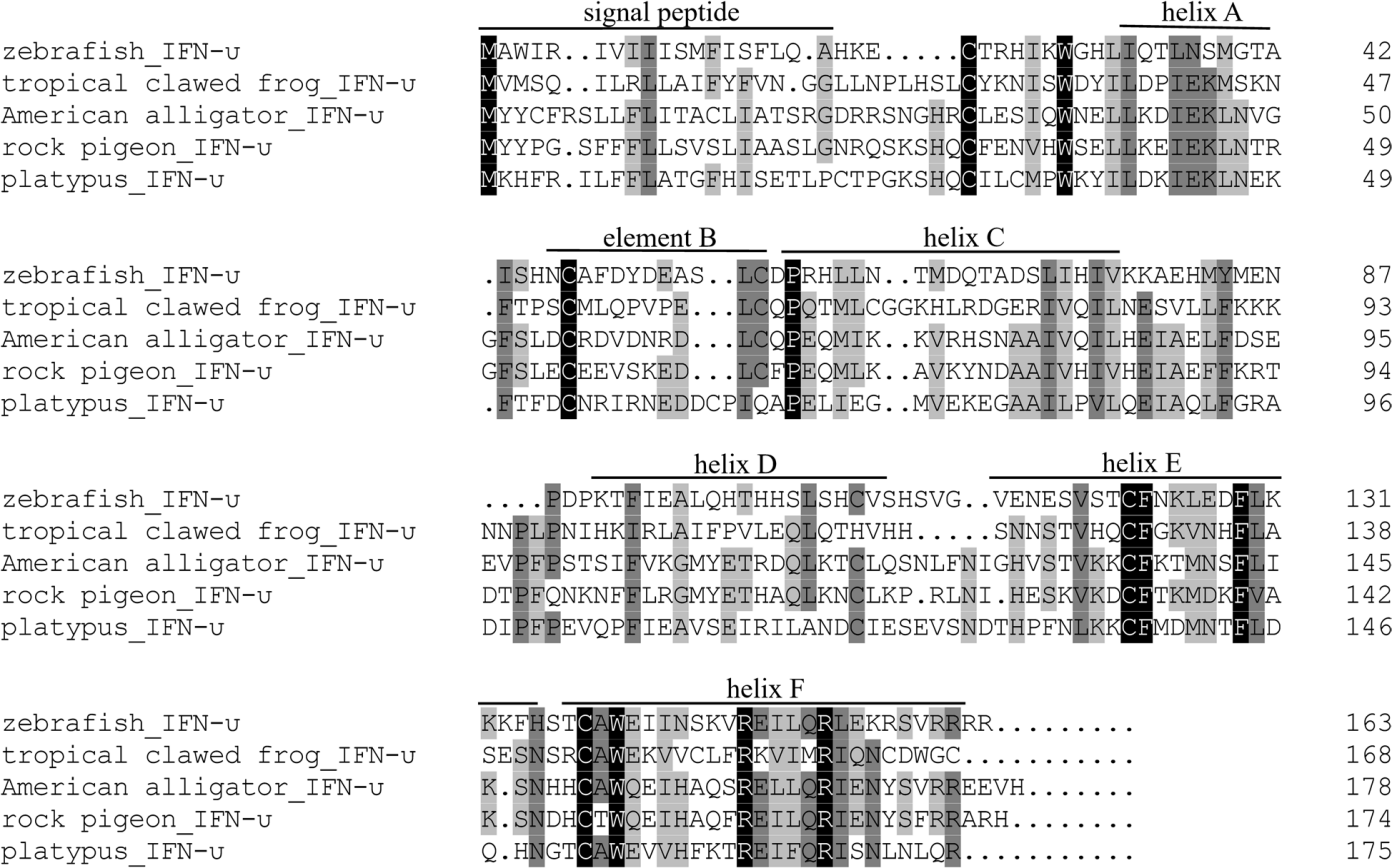
**Amino acid sequence alignment of IFN-υ from rock pigeon and other vertebrates**. Residue conservation is indicated by black, dark gray, and light gray shading, representing identical, highly similar, and weakly similar residues, respectively. Underlined segments correspond to the predicted signal peptide and the six helical domains (**A**–**F**). Gene locations or accession numbers are as follows: zebrafish_IFN-υ, MW547062; tropical clawed frog_IFN-υ, MW924835; American alligator_IFN-υ, NW_017707901.1: 15171479…15177353; rock pigeon_IFN-υ, PX776634; platypus_IFN-υ, NW_001794186.1: 7979635…7986399.

The rock pigeon (*Columba livia*) IFN-υ gene (GenBank accession number: PX776634) comprises 522 base pairs (bp), encoding a 174-amino acid (aa) protein with predicted molecular weight of ~20 kDa. Structural analysis revealed an N-terminal signal peptide (residues 1–21 aa) and a highly conserved multi-helix structure (residues 22–174 aa) (Figure 1). Phylogenetic reconstruction placed rock pigeon IFN-υ within a monophyletic avian clade, which was further nested within a broader vertebrate lineage comprising reptilian, primitive mammals, fish, and amphibian orthologs (Additional file 1).

**Figure 2:**
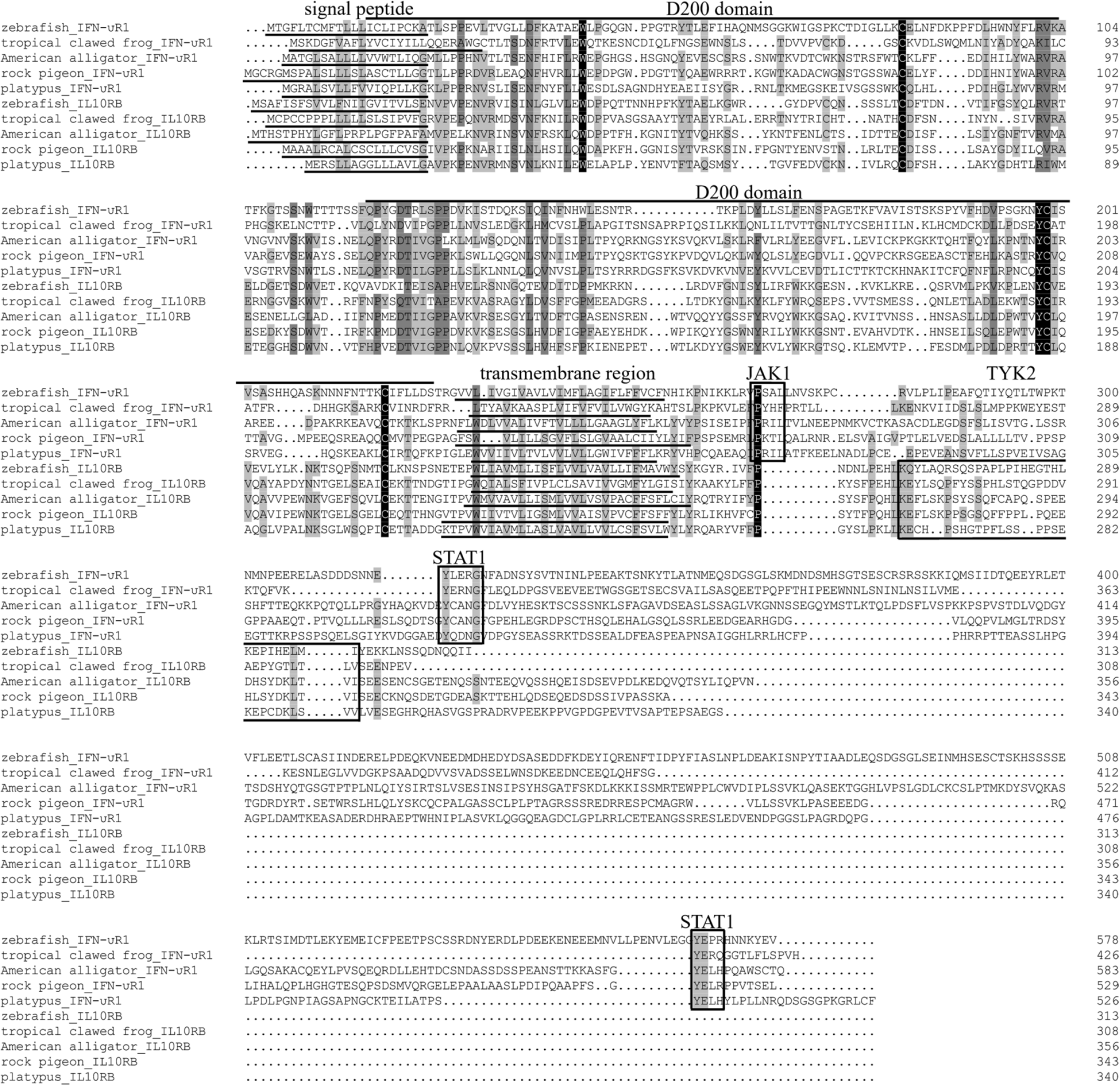
**Sequence alignment of rock pigeon IFN-υR1 with IL10RB and other vertebrate homologs**. Residue conservation is highlighted with black (identical), dark gray (highly similar), and light gray (low similarity). Predicted signal peptides and transmembrane domains are underlined. The D200 domain is marked by a solid line above the alignment. Boxes indicate potential intracellular binding sites for JAK1, TYK2, and STAT1. Reference sequences are as follows: zebrafish_IFN-υR1 (NP_001035443.1); tropical clawed frog_IFN-υR1 (XP_002940450.1); American alligator_IFN-υR1 (XP_019337453.1); rock pigeon_IFN-υR1 (XP_021145800.1); platypus_IFN-υR1 (NW_001794458.1: 8968090…8983283); zebrafish_IL10RB (NP_001124447.1); tropical clawed frog_IL10RB (NP_001165294.1); American alligator_IL10RB (XP_059576675.1); rock pigeon_IL10RB (XP_064922738.1); platypus_IL10RB (XP_028937522.1).

Protein sequence analysis of rock pigeon IFN-υR1 (XM_021290125.1) and IL10RB (XM_065066666.1) revealed the canonical domain architecture of type II cytokine receptors, consisting of an extracellular region with a predicted N-terminal signal peptide, two extracellular D200 domains, a transmembrane domain formed by a stretch of hydrophobic amino acids, and an intracellular region (Figure 2). Notably, the intracellular region of IFN-υR1 contains predicted binding motifs for JAK1 (PKTL) and STAT1 (YCANG and YELR) (Figure 2), while that of IL10RB harbors a predicted TYK2 binding motif (KEFLSKPPSGSQFFPPLPQEEHLSYDKLTVI) (Figure 2) homologous to the corresponding region of human IL10RB (KEFLGHPHHNTLLFFSFPLSDENDVFDKLSVI). Phylogenetic analysis demonstrated that rock pigeon IFN-υR1 and IL10RB were each clustered into distinct, well-supported clades with their respective orthologs from other vertebrate species (Additional file 1).

### Constitutive and induced expression of *IFN-υ*, *IFN-υR1*, and *IL10RB*

**Figure 3:**
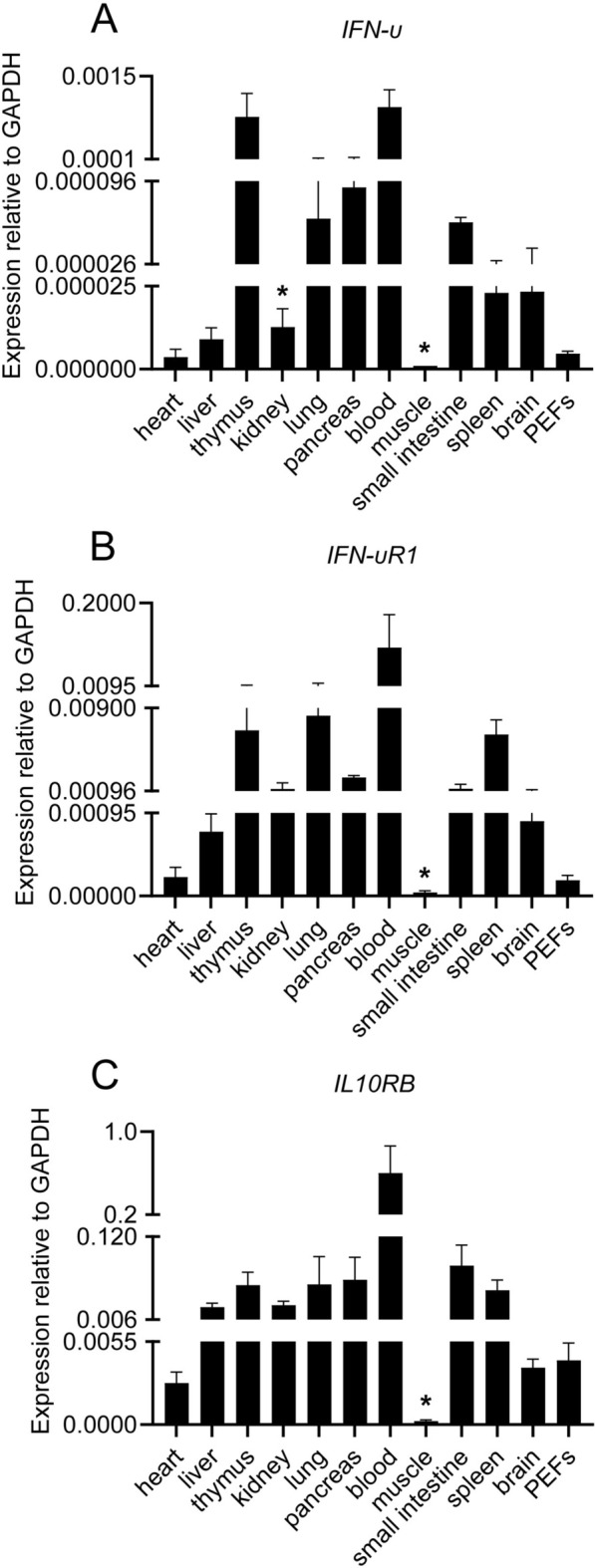
**Constitutive expression of rock pigeon IFN**-υ **and its receptor genes across organs/tissues**. **A**–**C** Background mRNA expression of *IFN*-υ (**A**), *IFN-υR1* (**B**), and *IL10RB* (**C**) was quantified by qRT-PCR in 11 organs/tissues (heart, liver, thymus, kidney, lung, pancreas, blood, muscle, small intestine, spleen, and brain) from healthy, untreated adult rock pigeons (*n* = 3), as well as in PEFs. Total RNA was extracted using the VAMNE Magnetic Cell/Tissue Total RNA Kit, followed by DNase I treatment and reverse transcription, and qRT-PCR was performed using SYBR Green dye on a QuantStudio 3 Real-Time PCR System. Gene expression was normalized to *GAPDH* and presented as mean ± SE. Statistical significance of differences among organs/tissues was evaluated by one-way ANOVA with Tukey's post hoc test; asterisks (*) denote significant differences (*P* < 0.05).

The basal expression profiles of *IFN-υ*, *IFN-υR1*, and *IL10RB* were examined by qRT-PCR in 11 organs/tissues (heart, liver, thymus, kidney, lung, pancreas, blood, muscle, small intestine, spleen, and brain) of healthy rock pigeons, as well as in PEFs, using GAPDH as internal reference. The results revealed constitutive yet distinct tissue-specific expression patterns for all three genes across the examined organs/tissues and PEFs (Figure 3A–C). *IFN-υ* expression was highest in blood and thymus, moderate in pancreas, lung, and small intestine, and lowest in heart and muscle (Figure 3A). The receptor genes *IFN-υR1* and *IL10RB* exhibited highly similar expression trends, with both showing the most pronounced expression in blood, maintaining relatively high levels in thymus, pancreas, and small intestine, and displaying minimal expression in heart, muscle, and PEFs (Figure 3B and C). Furthermore, *IL10RB* exhibited consistent moderate expression in liver, kidney, and spleen (Figure 3C).

**Figure 4:**
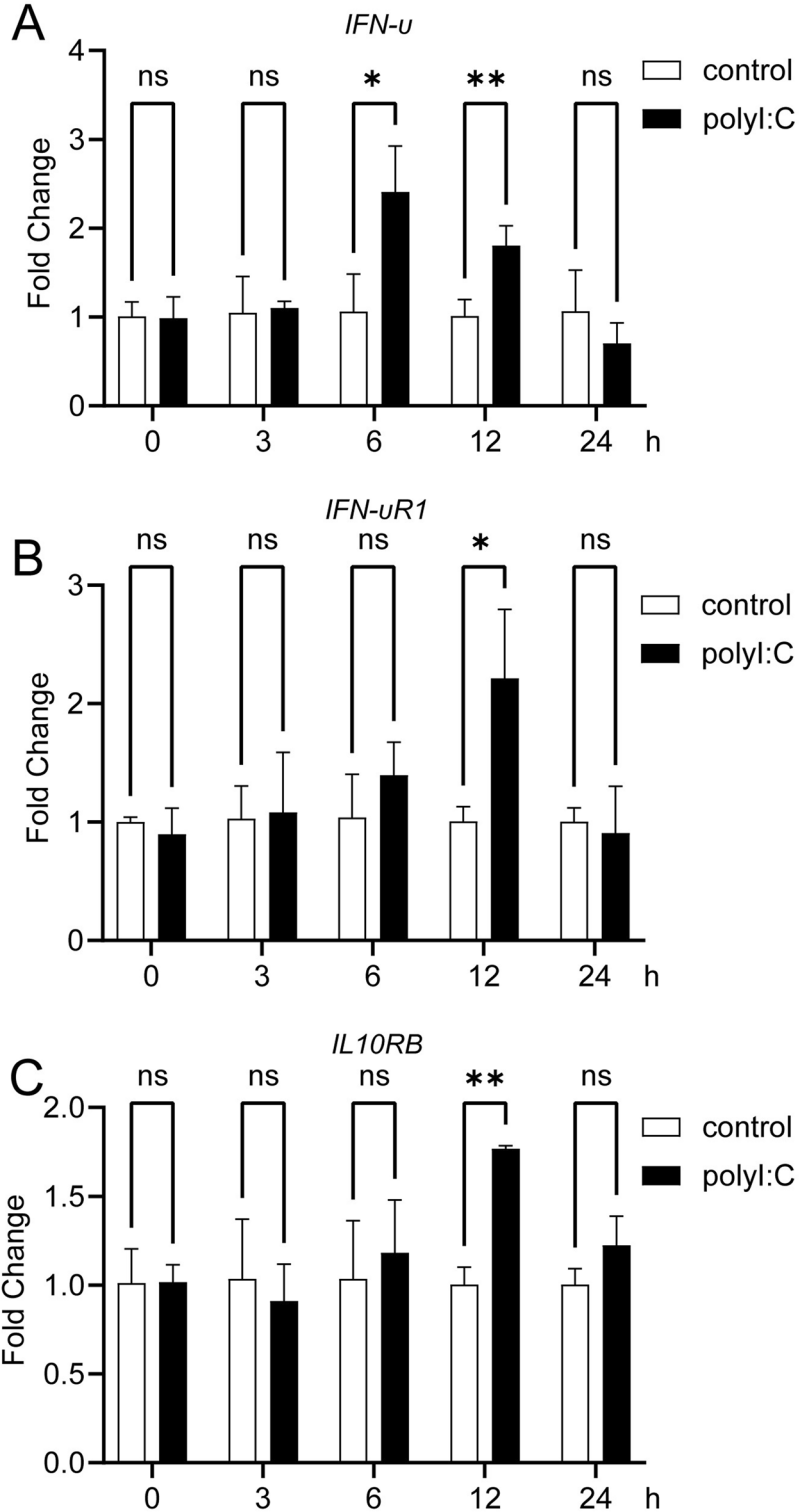
**Poly(I:C) stimulation upregulates the expression of IFN-υ and its receptors in rock pigeon liver leukocytes**. **A**–**C** Liver leukocytes isolated from healthy rock pigeons were seeded in 12-well plates and stimulated with poly(I:C) (2 μg/mL). Cells were harvested at the indicated time points (0, 3, 6, 12, and 24 h post-stimulation). Total RNA was extracted, reverse-transcribed, and subjected to qRT-PCR to determine the mRNA levels of *IFN-υ* (**A**), *IFN-υR1* (**B**), and *IL10RB* (**C**). Gene expression was normalized to *GAPDH*. Data are presented as the mean ± SE from three independent experiments. Student's *t*-test was used to compare the experimental group to the control group. Significance is denoted as follows: *P < 0.05, **P < 0.01, and ns for nonsignificant results.

To elucidate the response characteristics of the IFN-υ system to viral mimic stimulation, the expression dynamics of *IFN-υ*, *IFN-υR1*, and *IL10RB* in rock pigeon liver leukocytes was examined following poly(I:C) treatment. As shown in Figure 4A, *IFN-υ* expression was markedly induced after poly(I:C) stimulation, with obvious peaks observed at 6 h. The highest fold induction exceeded 2.4 folds at 6 h and remained around 1.8 fold at 12 h, before gradually declining by 24 h (Figure 4A). The receptor gene *IFN-υR1* was also inducible by poly(I:C), showing a relatively moderate upregulation which peaked at 12 h (approximately 2.2 folds), while fold changes at other time points were mostly below two folds (Figure 4B). Similarly, the expression of the co-receptor *IL10RB* was enhanced after stimulation, with a noticeable increase at 12 h (about 1.8 folds) and a relatively stable level of induction maintained throughout the time course (Figure 4C).

### Induction of immune-related genes by recombinant IFN-υ protein

**Figure 5:**
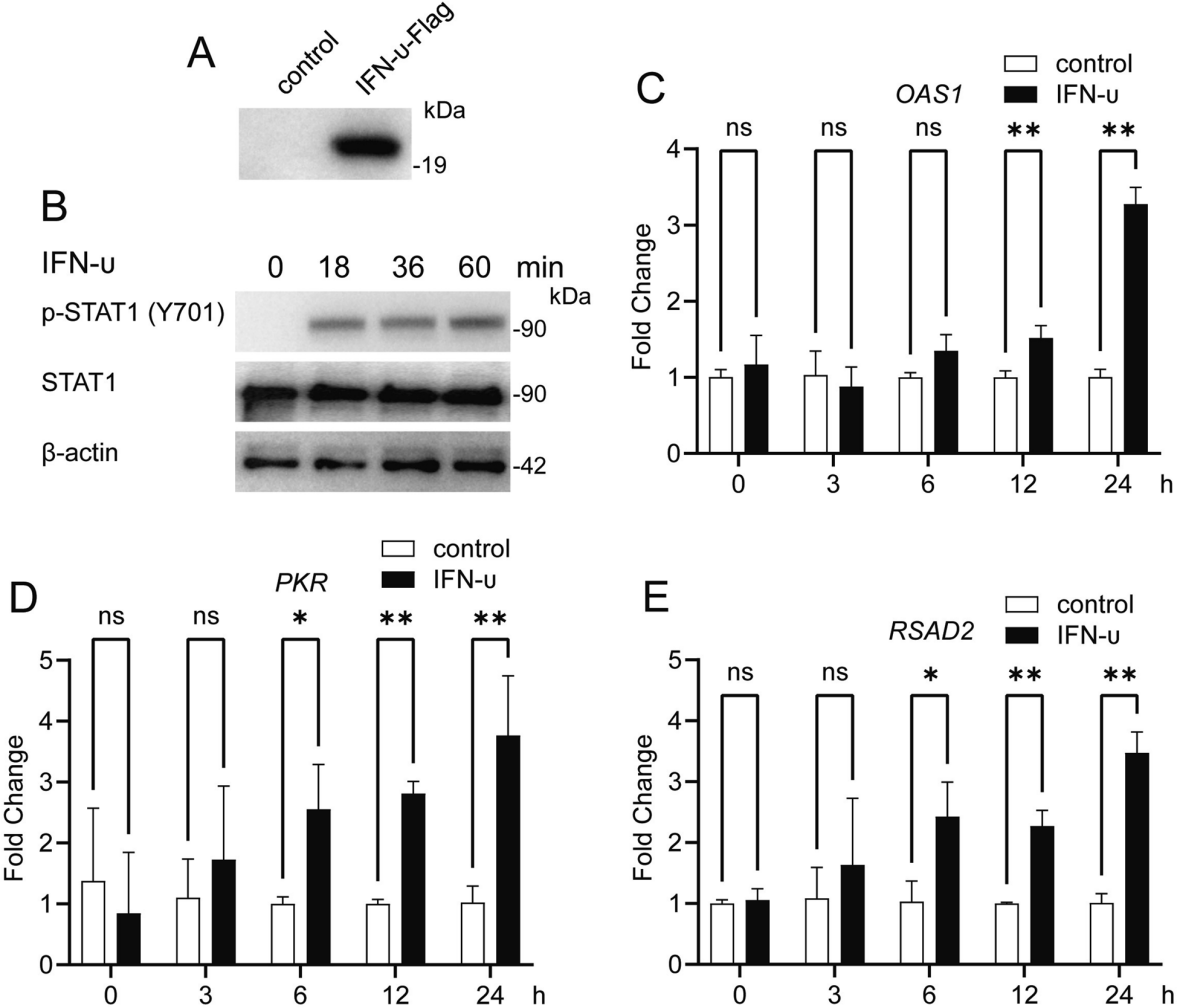
**The rock pigeon IFN-υ activates the JAK-STAT signaling pathway and induces ISG expression**. **A** Expression and detection of recombinant rock pigeon IFN-υ-Flag protein. HEK293T were transfected with a plasmid encoding rock pigeon IFN-υ-Flag. The cell culture supernatant was collected 48 h post-transfection. Samples of the supernatant were mixed with 5× SDS-PAGE loading buffer, denatured by heating at 100 °C for 10 min, and analyzed by Western blot using an anti-Flag antibody to detect the recombinant IFN-υ-Flag protein. **B** Rock pigeon IFN-υ activates the JAK-STAT signaling pathway. PEFs were stimulated with recombinant IFN-υ protein collected from transfected HEK293T (as in **A**). Cells were harvested at 0, 18, 36, and 60 min post-stimulation. Cell lysates were prepared using RIPA lysis buffer supplemented with protease and phosphatase inhibitors. Activation of the pathway was assessed by Western blot analysis of STAT1 phosphorylation (p-STAT1). **C**–**E** Rock pigeon IFN-υ induces transcriptional upregulation of downstream ISGs. PEFs were stimulated with recombinant IFN-υ protein and harvested at 0, 3, 6, 12, and 24 h post-stimulation. Total RNA was extracted and reverse-transcribed into cDNA, and the mRNA expression levels of representative ISGs (*OAS1*, *PKR*, and *RSAD2*) were analyzed by qRT-PCR. Data are presented as mean ± SE. Statistical significance was determined by Student's *t*-test (*P < 0.05, **P < 0.01, and ns for nonsignificant results).

To further validate the function of rock pigeon IFN-υ, primary rock pigeon liver leukocytes were treated with recombinant IFN-υ protein (Figure 5A). This treatment resulted in the activation of the JAK-STAT signaling pathway, as evidenced by the induced phosphorylation of STAT1 (Figure 5B). Subsequently, this signaling cascade led to the significant upregulation of a panel of ISGs, including *OAS1* (GenBank: XM_005508863.3), *PKR* (GenBank: XM_069852483.1), and *RSAD2* (GenBank: XM_021293639.2) (Figure 5C–E). In addition, IFN-υ stimulation significantly upregulated the expression of *IFN-υR1* and *IL10RB* in PEFs (Additional file 2). It is obvious that *IFN-υ* and its receptor genes are rapidly induced by poly(I:C), with *IFN-υ* exhibiting a stronger and earlier response, and rock pigeon IFN-υ is functionally active in activating the JAK-STAT signaling pathway and inducing ISG expression, indicating that this system plays an important regulatory role in antiviral innate immunity in rock pigeon.

### Functional dependence of rock pigeon IFN-υ on its receptor complex IFN-υR1/IL10RB

**Figure 6:**
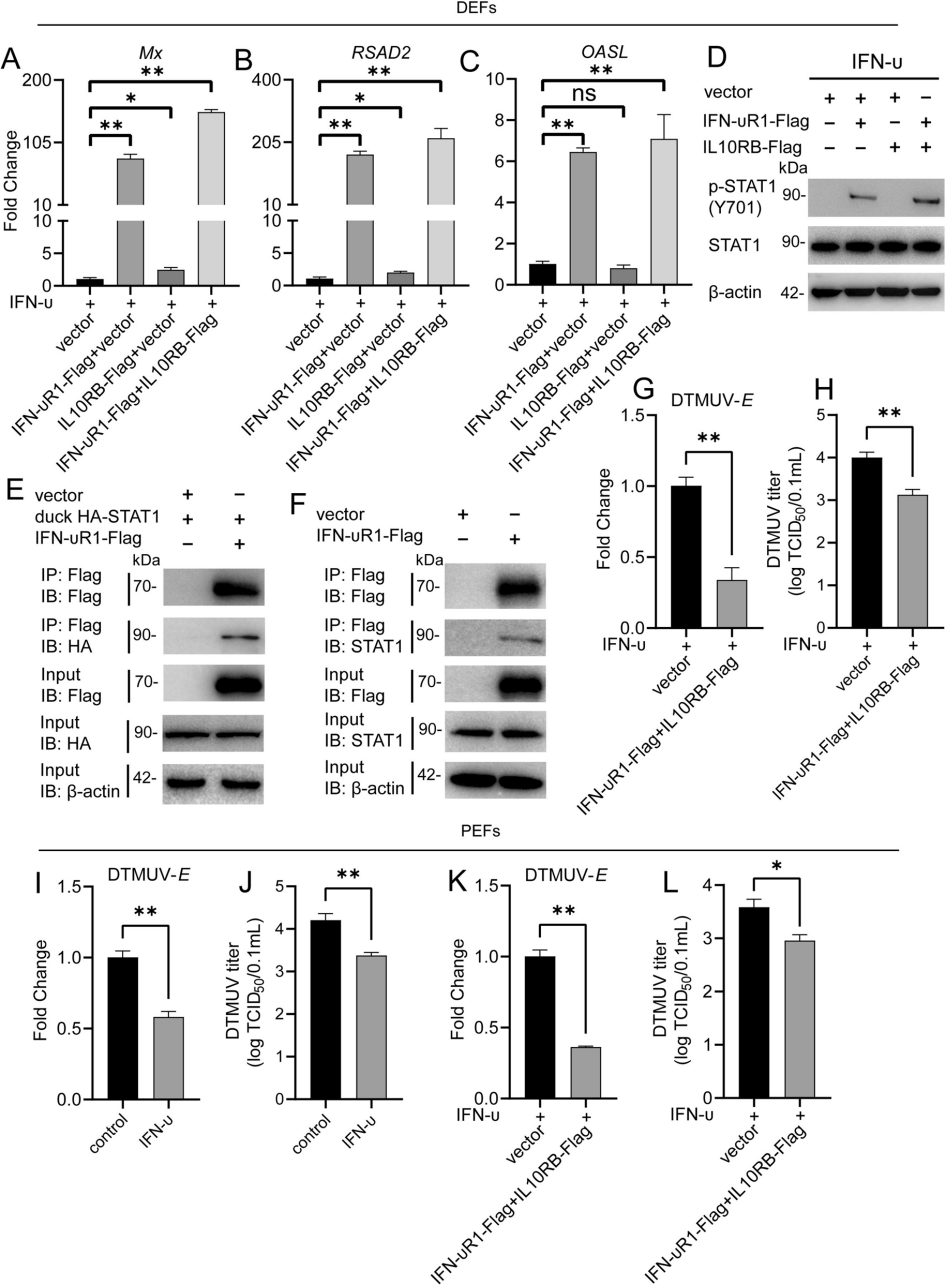
**Rock pigeon IFN-υ exerts antiviral functions through specific receptors IFN-υR1 and IL10RB**. **A**–**C** IFN-υ induces ISG expression via IFN-υR1 and IL10RB, as determined by qRT-PCR of *Mx* (**A**), *RSAD2* (**B**), and *OASL* (**C**) in DEFs with the indicated plasmid treatments and IFN-υ stimulation. **D** IFN-υ activates the JAK-STAT pathway via IFN-υR1 and IL10RB, as shown by Western blot of p-STAT1 in DEFs treated with indicated plasmids and IFN-υ. **E**, **F** Protein interaction between rock pigeon IFN-υR1 and duck STAT1 was assessed by Co-IP followed by Western blot. **G**, **H** IFN-υ inhibits DTMUV infection in DEFs via IFN-υR1 and IL10RB, as determined by qRT-PCR of DTMUV-*E* (**G**) and TCID₅₀ titration (**H**). **I**–**L** IFN-υ inhibits DTMUV infection in PEFs via IFN-υR1 and IL10RB, as determined by qRT-PCR of DTMUV-*E* and TCID_50_ titration at 40 h post-infection (hpi) in cells either treated directly with IFN-υ (**I**, **J**) or transfected with the receptor combination prior to IFN-υ stimulation (**K**, **L**). Statistical analysis was performed using Student’s *t*-test (*P < 0.05, **P < 0.01).

To determine whether rock pigeon IFN-υ exerts its function through its putative receptors IFN-υR1 and IL10RB, a receptor reconstitution assay was performed in DEFs. DEFs were co-transfected with expression plasmids for rock pigeon IFN-υR1-Flag, IL10RB-Flag, or both, along with vector control. Subsequent stimulation with recombinant IFN-υ protein revealed that the expression of *Mx*, *RSAD2*, and *OASL* (XM_038182224.2, KF483849.1, and KX255654.1, respectively) was significantly upregulated in cells expressing either IFN-υR1 or IL10RB alone, compared to vector-transfected controls (Figure 6A–C). Notably, the highest level of ISG induction was observed in DEFs co-expressing both IFN-υR1 and IL10RB, indicating a cooperative effect of the receptor complex (Figure 6A–C).

Consistently, treatment of IFN-υR1/IL10RB-co-expressing DEFs with IFN-υ resulted in the activation of the JAK-STAT pathway, as demonstrated by the phosphorylation of STAT1 (Figure 6D). Co-IP assays confirmed a physical interaction between rock pigeon IFN-υR1 and duck STAT1 (GenBank: XM_027460933.2), using both exogenous (Figure 6E) and endogenous (Figure 6F) protein sources. In addition, a protein interaction was also detected between rock pigeon IFN-υR1 and duck IL10RB (Additional file 3). Crucially, this IFN-υ-induced signaling via the reconstituted rock pigeon receptor complex conferred a potent antiviral state, significantly inhibiting the replication of DTMUV-*E* (Figure 6G) and substantially reducing viral titer in DEFs (Figure 6H).

It has been reported that pigeons can serve as host for DTMUV infection [31]. Thus, experiments on resistance to DTMUV infection were conducted to further verify the full functionality of rock pigeon IFN-υ in PEFs. The results showed that both treatment with recombinant IFN-υ protein alone (Figure 6I and J) and co-overexpression of IFN-υR1/IL10RB (Figure 6K and L) effectively suppressed DTMUV replication and reduced viral titers.

**Figure 7:**
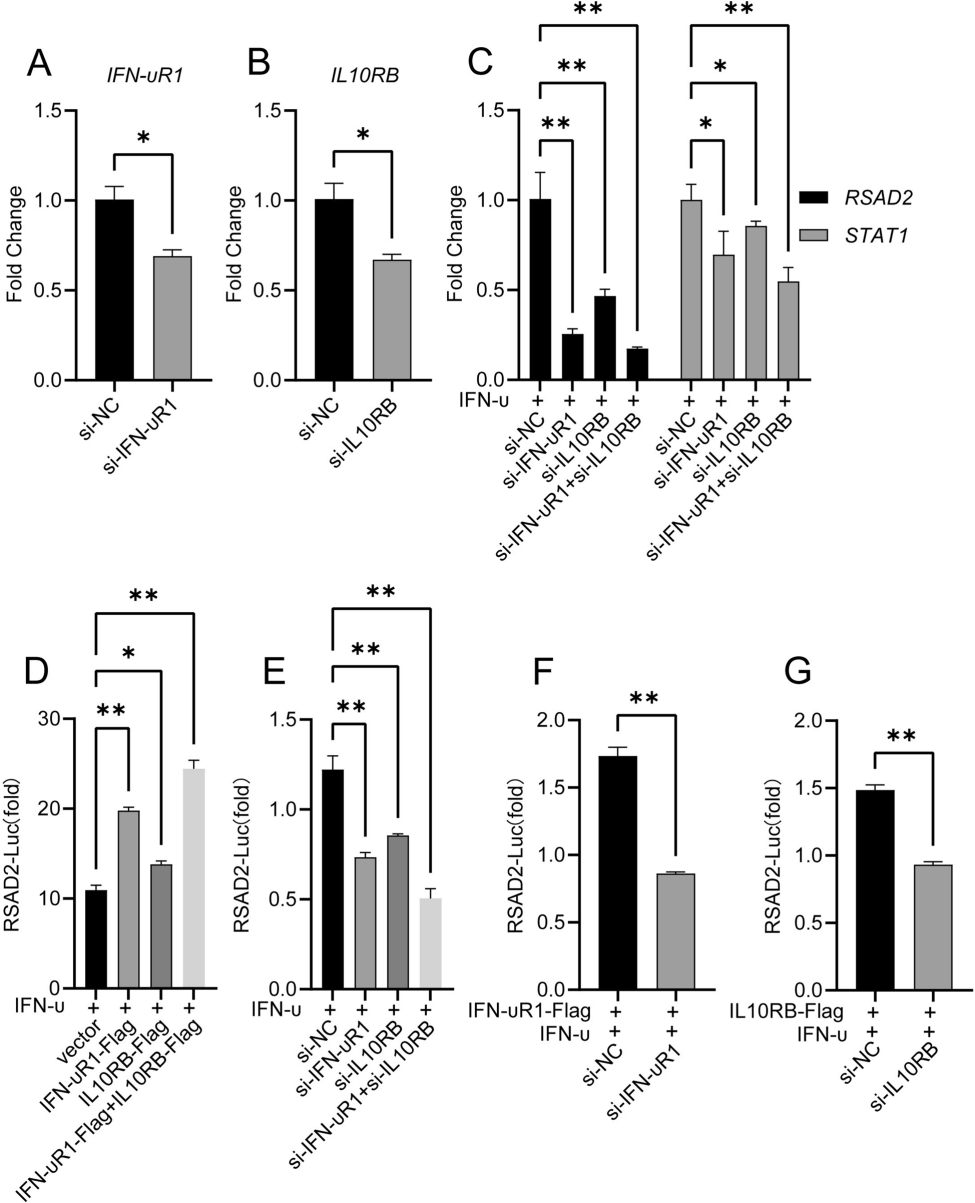
**Knockdown of rock pigeon IFN-υR1 or IL10RB attenuates IFN-υ-induced ISG expression.** **A**, **B** Validation of knockdown efficiency for rock pigeon IFN-υR1 and IL10RB by qRT-PCR. **C** Knockdown of IFN-υR1 and IL10RB reduced IFN-υ-induced *RSAD2* and *STAT1* expression, as measured by qRT-PCR. **D** Overexpression of IFN-υR1 and IL10RB enhances IFN-υ-induced *RSAD2* promoter activity. **E** Endogenous IFN-υR1 or IL10RB knockdown weakens the IFN-υ-mediated induction of the *RSAD2* promoter. **F**, **G** Knockdown abolishes the potentiating effect of overexpressed IFN-υR1 or IL10RB on IFN-υ-induced *RSAD2* promoter activity. Data are presented as mean ± SE from three independent experiments. *P < 0.05, **P < 0.01 (Student’s *t*-test).

To further confirm the functional requirement of IFN-υR1 and IL10RB for IFN-υ signaling, we performed siRNA-mediated knockdown of each receptor in PEFs. Transfection of specific siRNAs targeting IFN-υR1 or IL10RB significantly reduced the expression of the respective receptors (Figure 7A and B). Subsequent stimulation of these knockdown cells with recombinant rock pigeon IFN-υ protein resulted in a marked decrease in the upregulation of ISGs *RSAD2* and *STAT1* (XM_065068939.1) (Figure 7C), as measured by qRT-PCR. Consistently, dual-luciferase reporter assays demonstrated that the promoter activity of *RSAD2* was substantially attenuated upon IFN-υ stimulation in receptor-knockdown PEFs compared to control cells (Figure 7D–G). As a positive control, co-transfection of overexpression plasmids together with IFN-υ treatment markedly enhanced RSAD2 promoter activity (Figure 7D). Notably, after knockdown of endogenous IFN-υR1 or IL10RB, stimulation with IFN-υ resulted in significant reduction in promoter activity when both receptors were knocked down simultaneously compared with knockdown of each receptor gene alone (Figure 7E). Furthermore, following knockdown of exogenously overexpressed IFN-υR1 or IL10RB and subsequent IFN-υ treatment, RSAD2 promoter activity was also significantly suppressed (Figure 7F and G). These loss-of-function results provide direct molecular evidence that both IFN-υR1 and IL10RB are essential and specific components of the functional receptor complex for rock pigeon IFN-υ.

### Functional identification of key tyrosine residues in rock pigeon IFN-υR1 for type IV IFN signaling

**Figure 8:**
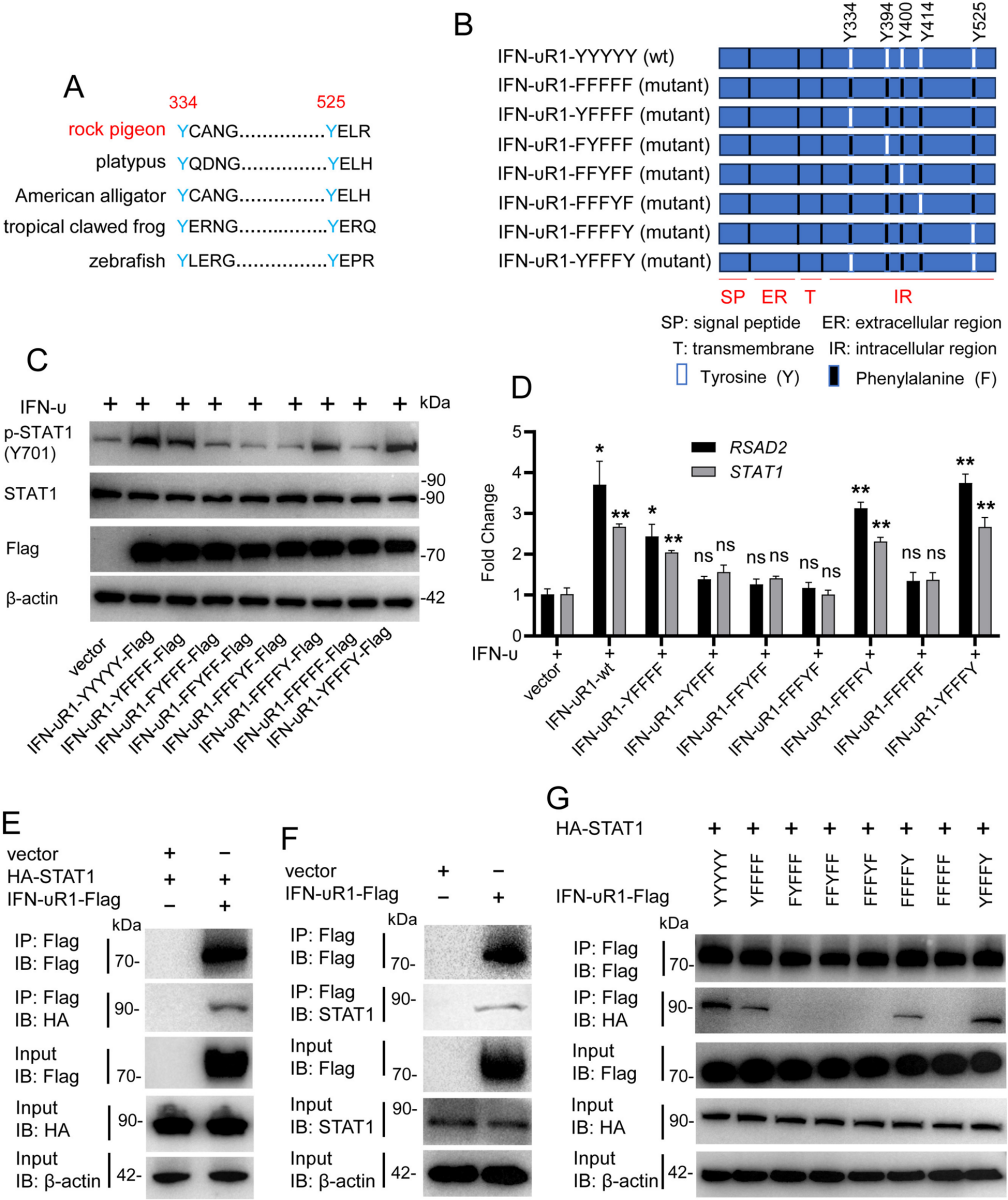
**Functional identification of conserved tyrosine residues in the intracellular domain of rock pigeon IFN-υR1. A** Two tyrosine residues are conserved in IFN-υR1 across vertebrates. In rock pigeon IFN-υR1, these correspond to Tyr_334_ and Tyr_525_. **B** Schematic representation of wild-type (wt) rock pigeon IFN-υR1 and its tyrosine mutant constructs. **C** Tyr_334_ and Tyr_525_ are critical for STAT phosphorylation. PEFs were transfected with vector, IFN-υR1-Flag (wt), or its mutant plasmids. At 24 h post-transfection, cells were stimulated with IFN-υ for 1 h, then lysed and analyzed by Western blot for p-STAT1. **D** Tyr_334_ and Tyr_525_ are also essential for IFN-υ-induced ISG transcription. Similarly treated PEFs were harvested 24 h after IFN-υ stimulation. The mRNA expression levels of *RSAD2* and *STAT1* were measured by qRT-PCR. **E**, **F** Co-IP assays confirm the protein–protein interaction between rock pigeon IFN-υR1 and STAT1. **G** Tyr_334_ and Tyr_525_ of rock pigeon IFN-υR1 are also involved in its interaction with STAT1, as assessed by Western blot.

The intracellular domains of class II cytokine receptors, including IFN receptors, often have conserved tyrosine residues as docking sites to recruit and activate STAT transcription factors. To identify such critical regulatory residues in the newly characterized type IV IFN receptor, a comparative sequence analysis of IFN-υR1 was performed from fish to primitive mammals. This analysis revealed two very highly conserved tyrosine residues within its intracellular region, namely Tyr_334_ and Tyr_525_ in rock pigeon; in addition, three other tyrosine residues (e.g., Tyr_394_, Tyr_400_, and Tyr_414_ in rock pigeon) were found to be conserved only in birds and reptiles (Figures 2 and 8A). To functionally assess these sites, corresponding point mutations were generated (Figure 8B). Functional analysis demonstrated that the presence of either Tyr_334_ or Tyr_525_ alone in rock pigeon IFN-υR1 is important for IFN-υ-induced STAT1 phosphorylation (Figure 8C) and the upregulation of *RSAD2* and *STAT1* (Figure 8D). Moreover, it is noteworthy that the simultaneous presence of both tyrosine residues (i.e., both Tyr_334_ and Tyr_525_ are retained) completely restored signaling (Figure 8C and D). In contrast, Tyr_394_, Tyr_400_, and Tyr_414_ in rock pigeon IFN-υR1 do not participate in IFN-υ signaling (Figure 8D); given their lineage-restricted conservation, they likely play species-specific regulatory roles.

To further determine whether these tyrosines were involved in the interaction between rock pigeon IFN-υR1 and STAT1, Co-IP assays were conducted. In PEFs co-overexpressing IFN-υR1-Flag and HA-tagged STAT1, as well as under conditions where only IFN-υR1-Flag was overexpressed and endogenous STAT1 was examined, a clear association was observed between wild-type IFN-υR1 and STAT1 (Figure 8E and F). Notably, point mutations targeting the conserved tyrosines (Y334F and Y525F) reduced this co-immunoprecipitation compared to the wild-type receptor; conversely, retention of these two tyrosine residues restored the binding ability (Figure 8G). Collectively, these results demonstrate that the conserved tyrosine residues in rock pigeon IFN-υR1 are essential for phosphorylation-dependent signal transduction, and also contribute to mediating or stabilizing the IFN-υR1-STAT1 protein–protein interaction.

## Discussion

Since its initial discovery in 2022, type IV IFN, i.e. IFN-υ, has been characterized in several species of vertebrates including teleost and cartilaginous fish, clawed frog, and mallard, with respect to its genomic presence, receptor architecture, signal transduction, and transcriptional regulation and function [3–5, 13–15, 30]. In addition to the identification of IFN-υ and its receptor subunits, IFN-υR1 and IL10RB, in rock pigeon, the recombinant IFN-υ was confirmed to confer a potent antiviral state against DTMUV in DEFs by engaging its receptor complex, and the two conserved tyrosine residues (Tyr_334_ and Tyr_525_) in the intracellular domain of IFN-υR1 are essential for interacting with STAT1 and activating the downstream JAK-STAT pathway, thereby driving ISG expression. The present study thus provides further information on the signaling of type IV IFN.

Type IV IFN (IFN-υ) is an evolutionarily conserved type of IFNs in vertebrates, and its signaling mechanism via the heterodimeric receptor complex IFN-υR1/IL10RB has been well defined and demonstrated to play a crucial role in inducing an antiviral state across a diverse range of vertebrate species [3, 5, 12, 14, 30]. In rock pigeon, IFN-υ protein also possesses typical structural features of type IV IFN, such as an N-terminal signal peptide and a conserved multi-helix domain, which aligns with the core structural definition of this protein family [5]. Its functional receptors, IFN-υR1 and IL10RB, contain the canonical domains of class  II cytokine receptors, including predicted binding sites for JAK kinases and STAT transcription factors, thereby forming the structural basis for activating the classic JAK-STAT signaling pathway [3, 5, 12]. It has been experimentally validated that IFN-υ interacts with its receptor complex by expressing receptors in xenogeneic cells as well as through the receptor knockdown [3, 5, 30]. In this study, the overexpression of IFN-υR1 alone exerted a certain dominant role in IFN-υ signaling in DEFs. This may be attributed to the lack of a conserved tyrosine residue (corresponding to Tyr_525_ in pigeons) on duck IFN-υR1, together with the structural conservation of the IFN-υ receptor system [5, 30], which enables the overexpressed IFN-υR1 to form a cross-species receptor complex with endogenous duck IL10RB, thereby producing a “complementation” effect and amplifying IFN-υ signaling. However, the ability of co-transfection of both IFN-υR1 and IL10RB in DEFs maximally activates STAT1 phosphorylation and induces ISG expression, which confirms the complex receptor units for IFN-υ in rock pigeon for the establishment of an effective antiviral state against DTMUV infection. The impaired transcription of ISGs and their promoter activity in receptor-specific siRNA knockdown experiments, together with the enhanced resistance to DTMUV infection upon the overexpression of the two receptor subunits in PEFs, further confirmed the essentiality of both IFN-υR1 and IL10RB in the type IV IFN signaling pathway from ligand-receptor binding and STAT1 activation to downstream effector gene expression.

The constitutive and inducible expression of IFNs and their related receptor components represents a conserved regulatory feature of the IFN system in vertebrates [7]. Consistent with this paradigm and with reports in other species [3, 4, 13–15], the IFN-υ transcripts in rock pigeon were most abundantly detected in immunologically active tissues such as blood and thymus, with its receptor genes showing highly correlated expression, as reported similarly in ducks [30]. The low expression of IFN-υR1 and IL10RB in liver and PEFs may be the main factor limiting IFN-υ signal transduction in rock pigeon. It has been reported that IFN-υ expression is upregulated following viral infection, poly(I:C) stimulation, or bacterial challenge [3, 12, 15]. Correspondingly in the present study, IFN-υ, IFN-υR1, and IL10RB were all significantly upregulated in rock pigeon liver leukocytes after poly(I:C) stimulation, indicating the role of the IFN-υ system in antiviral innate immune response.

It is well characterized that specific motifs in members of the class II cytokine receptor family contain conserved tyrosine residues in intracellular domains as docking sites to recruit and activate STAT transcription factors, a mechanism well established in type I, II, and III IFN receptors [18, 19, 21, 32]. For instance, Tyr_466_ on the type I IFN receptor subunit IFNAR1 serves as the primary docking site for recruiting STAT2 [20, 23], while Tyr_337_ and Tyr_512_ on IFNAR2c are responsible for recruiting both STAT1 and STAT2 [23]. In the type II IFN receptor IFNGR1, Tyr_440_ is an absolutely essential core docking site for STAT1 activation, and its mutation may result in complete abolishment in IFN-γ signaling [24]. Similarly, in type III IFN signaling, both STAT1 and STAT2 can dock at Tyr_517_ of the specific receptor subunit IFN-λR1, thereby initiating ISGF3-dependent antiviral gene expression [25]. These conserved phosphotyrosine residues within the STAT docking motifs have been experimentally verified [33–35]. So far, cumulative evidence as presented in this study has indicated that type IV IFN signaling relies on a same pathway [5, 12–15, 30], but the specific tyrosine residues in the intracellular domain of its specific receptor, IFN-υR1, has been revealed as STAT docking sites. Through evolutionary sequence analysis and site-directed mutagenesis, two residues, Tyr_334_ and Tyr_525_, in the intracellular domain of IFN-υR1 were verified to be essential for IFN-υ to activate downstream signaling and to induce ISG expression, and these two residues can be considered as the docking sites for recruiting STAT proteins (such as STAT1). The possible functional roles of the Tyr_334_ and Tyr_525_ residues in signal transduction and induction of ISG expression should be of interest in both comparative and evolutionary research on IFN-υ signaling in avian and also in piscine species.

In conclusion, the antiviral signaling cascade triggered by IFN-υ in rock pigeon was delineated via the two residues, Tyr_334_ and Tyr_525_, in the intracellular domain of IFN-υR1 for the docking of STAT1, leading to the phosphorylation of STAT1 and subsequent ISG expression. The results concerning the phosphotyrosine docking sites provides fundamental molecular and evolutionary insight into the conserved architecture of type IV IFN signaling.

## Supplementary Information

**Additional file 1: Phylogenetic analysis of IFN-υand its receptor subunitsin vertebrates**. The neighbor-joining trees were constructed based on the amino acid sequences of IFN-υ and the extracellular domains of its receptor subunits using the MAGE 11.0 program, with 1000 bootstrap replicates. Gene locations or accession numbers of the IFN-υ used for phylogenetic reconstruction are listed as follows: rock pigeon_IFN-υ, PX776634; duck_IFN-υ, OR369727; chicken_IFN-υ, CM000094.5: 11425652...11429077; American alligator_IFN-υ, NW_017707901.1: 15171479...15177353; painted turtle_IFN-υ, NC_024222.1: 3879779...3885334; platypus_IFN-υ, NW_001794186.1: 7979635...7986399; Australian echidna_IFN-υ, NC_052078.1:11991062...11998896; green anole_IFN-υ, UCL51438.1; common lizard_IFN-υ, NC_048614.1:14907892...14913259; African clawed frog_IFN-υ, MW924834; tropical clawed frog_IFN-υ, MW924835; zebrafish_IFN-υ, MW547062;channel catfish_IFN-υ, CM004414.1: 31816095...31812928. GenBank accession numbers for the receptor subunits are given below: chicken_IL10RB, NP_990188.1; duck _IL10RB, NP_001297340.1; rock pigeon_IL10RB, XP_064922738.1; American alligator_IL10RB, XP_059576675.1; platypus_ IL10RB, XP_028937522.1; Australian echidna_IL10RB, XP_038622203.1; mouse_IL10RB, NP_032375.2; human_IL10RB, NP_000619.3; green anole_IL10RB, NP_001315154.1; African clawed frog_IL10RB, NP_001087014.1; tropical clawed frog_IL10RB, NP_001165294.1; channel catfish_IL10RB, XP_017326115.1; grass carp_IL10RB, AHJ11133.1; zebrafish_IL10RB, NP_001077337.1; common carp_IL10RB, XP_018965682.1; zebrafish_IFN-υR1, NP_001035443.1;common carp_ IFN-υR1, XP_018939179.1; channel catfish_IFN-υR1, XP_017315324.1; gar_IFN-υR1, XP_015193079.1; African clawed frog_IFN-υR1, XP_018085133.1; tropical clawed frog_IFN-υR1, XP_002940450.1; green anole_IFN-υR1, XP_008119338.1; platypus_IFN-υR1, NW_001794458.1: 8968090...8983283; Australian echidna_IFN-υR1, XP_038604300.1; American alligator_IFN-υR1, XP_019337453.1; painted turtle_IFN-υR1, XP_005301412.1; chicken_IFN-υR1, XP_015133867.1; rock pigeon_IFN-υR1, XP_021145800.1; duck_IFN-υR1, NW_004677101.1: 470841...475790. Figure 2. The rock pigeon IFNυ induced IFNυR1 and IL10RB expression in PEFs.PEFs were seeded in a 24-well plate overnight and then incubated with recombinant IFNυ protein for 0, 3, 6, 12, and 24 h. qRTPCR was used to detect *IFNυR1*and *IL10RB*mRNA expression levels. Statistical significance was determined by Student's test. Figure 3. Rock pigeon IFN-υR1 interacted with duck IL10RB. DEFs were co-transfected with rock pigeon IFNυR1Flag and duck IL10RBGFP. At 48 h post-transfection, cell lysates were immunoprecipitated with anti-Flag beads. The immunoprecipitates were subjected to SDS-PAGE and Western blotting using anti-GFP or anti-Flag antibodies, respectively.

**Additional file 2: The rock pigeon IFN-υ induced IFN-υR1 and IL10RB expression in PEFs.** (**A and B**) PEFs were seeded in a 24-well plate overnight and then incubated with recombinant IFN-υ protein for 0, 3, 6, 12, and 24 h. qRT-PCR was used to detect *IFN-υR1* (**A**) and *IL10RB* (**B**) mRNA expression levels. Statistical significance was determined by Student's t-test (* *p* < 0.05, ** *p* < 0.01; ns, not significant).

**Additional file 3. Rock pigeon IFN-υR1 interacted with duck IL10RB.**DEFs were co-transfected with rock pigeon IFN-υR1-Flag and duck IL10RB-GFP. At 48 h post-transfection, cell lysates were immunoprecipitated with anti-Flag beads. The immunoprecipitates were subjected to SDS-PAGE and western blotting using anti-GFP or anti-Flag antibodies, respectively.

## Data Availability

No datasets were generated or analyzed during the current study.
