# Supplementary material for: Characterization of the rock pigeon type IV interferon system reveals conserved tyrosine-based signaling via the IFN-υR1/IL10RB complex
Source: Vet Res. 2026 Aug 12;57:146. doi: 10.1186/s13567-026-01823-w (PMC13466502; doi:10.1186/s13567-026-01823-w)
Supplement: Supplementary file 1 [file 13567_2026_1823_MOESM1_ESM.zip › Additional file 1/Additional file 1 legend.docx]

**Additional file 1. Phylogenetic analysis of IFN-υ (A) and its receptor subunits (B) in vertebrates.** The neighbor-joining trees were constructed based on the amino acid sequences of IFN-υ and the extracellular domains of its receptor subunits using the MAGE 11.0 program, with 1,000 bootstrap replicates. Gene locations or accession numbers of the IFN-υ used for phylogenetic reconstruction are listed below: rock pigeon_IFN-υ, PX776634; duck_IFN-υ, OR369727; chicken_IFN-υ, CM000094.5: 11425652...11429077; American alligator_IFN-υ, NW_017707901.1: 15171479...15177353; painted turtle_IFN-υ, NC_024222.1: 3879779...3885334; platypus_IFN-υ, NW_001794186.1: 7979635...7986399; Australian echidna_IFN-υ, NC_052078.1:11991062...11998896; green anole_IFN-υ, UCL51438.1; common lizard_IFN-υ, NC_048614.1:14907892...14913259; African clawed frog_IFN-υ, MW924834; tropical clawed frog_IFN-υ, MW924835; zebrafish_IFN-υ, MW547062;channel catfish_IFN-υ, CM004414.1: 31816095...31812928. GenBank accession numbers for the receptor subunits are given below: chicken_IL10RB, NP_990188.1; duck _IL10RB, NP_001297340.1; rock pigeon_IL10RB, XP_064922738.1; American alligator_IL10RB, XP_059576675.1; platypus_ IL10RB, XP_028937522.1; Australian echidna_IL10RB, XP_038622203.1; mouse_IL10RB, NP_032375.2; human_IL10RB, NP_000619.3; green anole_IL10RB, NP_001315154.1; African clawed frog_IL10RB, NP_001087014.1; tropical clawed frog_IL10RB, NP_001165294.1; channel catfish_IL10RB, XP_017326115.1; grass carp_IL10RB, AHJ11133.1; zebrafish_IL10RB, NP_001077337.1; common carp_IL10RB, XP_018965682.1; zebrafish_IFN-υR1, NP_001035443.1;common carp_ IFN-υR1, XP_018939179.1; channel catfish_IFN-υR1, XP_017315324.1; gar_IFN-υR1, XP_015193079.1; African clawed frog_IFN-υR1, XP_018085133.1; tropical clawed frog_IFN-υR1, XP_002940450.1; green anole_IFN-υR1, XP_008119338.1; platypus_IFN-υR1, NW_001794458.1: 8968090...8983283; Australian echidna_IFN-υR1, XP_038604300.1; American alligator_IFN-υR1, XP_019337453.1; painted turtle_IFN-υR1, XP_005301412.1; chicken_IFN-υR1, XP_015133867.1; rock pigeon_IFN-υR1, XP_021145800.1; duck_IFN-υR1, NW_004677101.1: 470841...475790.
