# Supplementary material for: Characterization of the rock pigeon type IV interferon system reveals conserved tyrosine-based signaling via the IFN-υR1/IL10RB complex
Source: Vet Res. 2026 Aug 12;57:146. doi: 10.1186/s13567-026-01823-w (PMC13466502; doi:10.1186/s13567-026-01823-w)
Supplement: Supplementary file 2 [file 13567_2026_1823_MOESM2_ESM.zip › Additional file 2/Additional file 2 legend.docx]

**Additional file 2.** **The rock pigeon IFN υ induced IFN υR1 and IL10RB expression in PEFs.** (A and B) PEFs were seeded in a 24 well plate overnight and then incubated with recombinant IFN υ protein for 0, 3, 6, 12, and 24 h. qRT PCR was used to detect IFN υR1 (A) and IL10RB (B) mRNA expression levels. Statistical significance was determined by Student's t test (* *p* < 0.05, ** *p* < 0.01; ns, not significant).
