# Supplementary material for: Characterization of the rock pigeon type IV interferon system reveals conserved tyrosine-based signaling via the IFN-υR1/IL10RB complex
Source: Vet Res. 2026 Aug 12;57:146. doi: 10.1186/s13567-026-01823-w (PMC13466502; doi:10.1186/s13567-026-01823-w)
Supplement: Supplementary file 3 [file 13567_2026_1823_MOESM3_ESM.zip › Additional file 3/Additional file 3 legend.docx]

**Additional file 3. Rock pigeon IFN-υR1 interacted with duck IL10RB.** DEFs were co‑transfected with rock pigeon IFN‑υR1‑Flag and duck IL10RB‑GFP. At 48 h post‑transfection, cell lysates were immunoprecipitated with anti‑Flag beads. The immunoprecipitates were subjected to SDS‑PAGE and western blotting using anti‑GFP or anti‑Flag antibodies, respectively.
